# Cadaverine Production From L-Lysine With Chitin-Binding Protein-Mediated Lysine Decarboxylase Immobilization

**DOI:** 10.3389/fbioe.2020.00103

**Published:** 2020-03-03

**Authors:** Ning Zhou, Alei Zhang, Guoguang Wei, Sai Yang, Sheng Xu, Kequan Chen, Pingkai Ouyang

**Affiliations:** State Key Laboratory of Materials-Oriented Chemical Engineering, College of Biotechnology and Pharmaceutical Engineering, Nanjing Tech University, Nanjing, China

**Keywords:** cadaverine, lysine decarboxylase, chitin-binding domain, immobilization, chitin

## Abstract

Lysine decarboxylase (CadA) can directly convert L-lysine to cadaverine, which is an important platform chemical that can be used to produce polyamides. However, the non-recyclable and the poor pH tolerance of pure CadA hampered its practical application. Herein, a one-step purification and immobilization procedure of CadA was established to investigate the cadaverine production from L-lysine. Renewable biomass chitin was used as a carrier for lysine decarboxylase (CadA) immobilization via fusion of a chitin-binding domain (ChBD). Scanning electron microscopy, laser scanning confocal microscopy, fourier transform infrared spectra, elemental analysis, and thermal gravimetric analysis proved that the fusion protein ChBD-CadA can be adsorbed on chitin effectively. Furthermore, the fusion protein (ChBD-CadA) existed better pH stability compared to wild CadA, and kept over 73% of the highest activity at pH 8.0. Meanwhile, the ChBD-CadA showed high specificity toward chitin and reached 93% immobilization yield within 10 min under the optimum conditions. The immobilized ChBD-CadA (I-ChBD-CadA) could efficiently converted L-lysine at 200.0 g/L to cadaverine at 135.6 g/L in a batch conversion within 120 min, achieving a 97% molar yield of the substrate L-lysine. In addition, the I-ChBD-CadA was able to be reused under a high concentration of L-lysine and retained over 57% of its original activity after four cycles of use without acid addition to maintain pH. These results demonstrate that immobilization of CadA using chitin-binding domain has the potential in cadaverine production on an industrial scale.

## Graphical Abstract

One-step purification and immobilization of lysine decarboxylase for converting L-lysine to cadaverine via fusion of chitin binding domain.

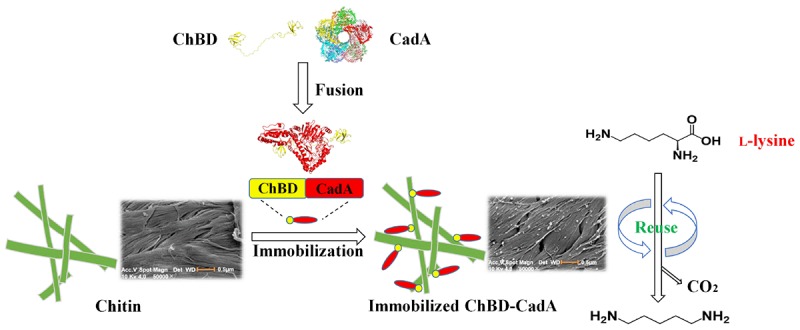

## Introduction

Polyamides (PA) are an essential polymer and widely used in engineering plastics, sportswear, sutures and catheters, owing to the excellent mechanical and thermal strength (Park et al., 2014; Jiang and Loos, 2016; Kim et al., 2018). Polyamides are mostly produced from chemicals extracted from fossil fuels (6.6 million tons per year), which contributes to the greenhouse effect and serious environmental pollution (Hong et al., 2004; Kind et al., 2011). Thus, environmentally friendly PA synthetic procedures have drawn increasing attention (Wang et al., 2018).

Cadaverine (1,5-diaminopentane), a bio-based chemical, can be combined with various bio-based diacids for the production of fully bio-based PAs, such as PA 5.4, PA 5.6, PA 5.10, and PA 5.12, which exhibit properties consistent with petroleum-based PAs (Buschke et al., 2011; Jiang and Loos, 2016; Seo et al., 2016).

To date, the bio-production of cadaverine has mainly been achieved by microbial fermentation and whole cell conversion from renewable resources. Microbial fermentation using engineered *E. coli* (Qian et al., 2011) and *C. glutamicum* (Kind et al., 2010; Kim et al., 2018) is a promising approach, which can produce relatively high yields and results in less impact on the environment. Whole-cell overexpressed lysine decarboxylase (LdcC or CadA) used as a catalyst is another attractive and greener approach (Park et al., 2015; Kloss et al., 2018); this method has the capability of producing cadaverine with high yields and high efficiency, and is environmentally friendly. In our previous study, recombinant *E. coli* overexpressing CadA was able to produce cadaverine at concentration of 228.0 g/L, which was the highest concentration reported to date (Ma et al., 2015b). In addition, biocatalysis using pure enzyme is also an efficient and well-established approach. However, the increase of pH and accumulation of product cadaverine during the reaction process could cause inactivation of CadA (Ma et al., 2017). Further, non-recyclable of free enzyme increases the cost, which inhibits its practical application (Singh and Kayastha, 2012; Guo et al., 2018).

Immobilization is an efficient method to enhance stability and reusability of enzymes, and this method allows for the easy separation of products (Liang et al., 2020). Several literatures involving immobilization of CadA for cadaverine production have been reported, while the loss of enzyme active and the low immobilization efficiency were observed (Seo et al., 2016; Park et al., 2017). Immobilization of enzymes via affinity adsorption is now recognized as a favorable method due to a simple and mild preparation procedure, strong binding affinity, and proper

exposure of the enzyme active site (Chiang et al., 2009). This method is also capable of simultaneous enzyme purification and immobilization.

The chitin-binding domain (ChBD) is a portion of some chitin hydrolysis enzymes and can specifically bind to chitin (the second most abundant natural polysaccharide after cellulose) via an affinity tag, which makes ChBD a popular choice for enzyme immobilization via protein fusion (Hashimoto et al., 2000; Simsek et al., 2013). ChBD has been employed to immobilize trehalose, levansucrase, β-galactosidase, and heparinase on chitin with excellent enzymatic outcomes (Chiang et al., 2009; Pham et al., 2017; Xu et al., 2017; Jiang et al., 2018).

In our previous study, a multi-functional chitinase (*Cm*Chi1) that contained two ChBDs from the bacterium *Chitinolyticbacter meiyuanensis* SYBC-H1 was expressed and characterized and showed excellent affinity toward the substrate chitin (Zhang et al., 2018). Here, we investigated cadaverine production from L-lysine by CadA immobilization on chitin based on fusion with ChBD from *Cm*Chi1. The characteristics and immobilization of the ChBD-CadA fusion protein were studied. In addition, repeated utilization of immobilized ChBD-CadA was also investigated.

## Materials and Methods

### Chemicals

L-Lysine hydrochloride, cadaverine, and chitin were purchased from Sigma-Aldrich (Shanghai, China). The molecular reagents were purchased from Takara Bio (Dalian, China). Other analytical grade chemicals and solvents were purchased from local suppliers.

### Strains, Plasmid, and Primers

The previously constructed plasmids pETDuet-*CadA* (Ma et al., 2015a), pET28a(+)-Cmchi1 (Zhang et al., 2018), and pET28a(+)-*gfp* were used as templates for cloning of the CadA gene, chitin binding domain (ChBD) gene, and green fluorescent protein (GFP) gene, respectively. The plasmid pETDuet-*CadA* was used as the expression vector for gene fusion. *E. coli* DH5α and *E. coli* BL21(DE3) cells (Novagen Co., Shanghai, China) were used as cloning and expression host, respectively, which were cultivated in Luria-Bertani (LB) broth or on 2% agar plates containing 50 μg/mL kanamycin.

Oligonucleotide primers used for PCR amplification were designed using Snap GeneTM 1.1.3 software^1^ and synthesized by Genscript Biotech (Nanjing, China). The forward and reverse primers containing endonuclease restriction sites used in this study are listed in Supplementary Table S1.

### Genes Cloning and Recombinant Plasmid Construction

PCR amplification with DNA polymerase was performed in a 50 μL reaction system and included 4 μL dNTPs, 0.5 μL of each primer, 1 μL fast Pfu DNA polymerase, 1 μL plasmid template, and the remaining volume was sterile water. The PCR amplification conditions were 95°C for 2 min, followed by 30 cycles of 95°C for 20 s, 53°C for 20 s, 72°C for 20 s; the extension procedure was carried out at 72°C for 5 min.

To construct the expression plasmid of the fusion gene *ChBD-CadA*, the fragment of *CadA* from plasmid pETDuet-*CadA* was amplified with DNA polymerase using primers F1 and R1. The fragment of *ChBD* from plasmid pET28a (+)-*ChBD* was amplified with the primers F3 and R3. The *ChBD* products were purified with the TIANquick MiDi Purification Kit and then digested using *Bam*HI and *Hin*dIII restriction enzymes and inserted into the *Bam*HI and *Hin*dIII sites of pET28a (+) expression plasmid to obtain the recombinant plasmid pET28a (+)-*ChBD*. The purified *CadA* product was digested using *Hin*dIII and *Not*I and inserted downstream of *ChBD* in pET28a (+)*-ChBD* to obtain the recombinant plasmid pET28a (+)-*ChBD-CadA.*

The construction of expression plasmid of fusion gene *CadA-ChBD* was the same as that for pET28a (+)-*ChBD-CadA.* The *CadA* fragment was amplified with primers F2 and R2 and digested using *Nco*I and *Bam*HI and inserted upstream of *ChBD* in pET28a (+)*-ChBD* to obtain the recombinant plasmid pET28a (+)-*CadA-ChBD.*

For construction of the expression plasmid for pET29a (+)-*gfp -ChBD-CadA*, the *gfp* fragment from pET28a(+)-*gfp* was amplified using primers F4 and R4, and then the purified PCR product was double digested by *Nde*I and *Bam*HI and ligated upstream of *ChBD-CadA* plasmid pET28a (+)-*ChBD-CadA*, resulting in plasmid pET28a (+)-*gfp-ChBD-CadA*. The transformants were characterized via colony PCR tests and sequenced by Genscript Biotech (Nanjing, China).

### Expression and Preparation of CadA, ChBD-CadA, and GFP-ChBD-CadA

The recombinant plasmids were transformed into *E. coli* BL21 (DE3) cells, and colonies were picked from agar plates and incubated in 100 mL fresh LB medium containing 50 μg/mL kanamycin) in a 500 mL shake flask at 37°C for 8–10 h with shaking at 200 rpm. Then, 20 mL of the pre-culture was used as the seed culture and was inoculated in a 1.4-L INFORS HT Multifors fermenter (Infors Biotechnology Co., Ltd., Beijing, China) containing 1 L LB medium and 50 μg/mL kanamycin at 37°C, with an aeration ratio of 1 vvm (vessel volume per minute) and agitation speed of 250 rpm, until the optical density at 600 nm (OD_600_) reached 0.6–0.8. The recombinant CadA, ChBD-CadA, and GFP-ChBD-CadA were induced at 37°C, 30°C, and 18°C, respectively, with a final concentration of 0.1 mM isopropyl-β-d-thiogalactopyranoside (IPTG) for 20 h. Consequently, the harvested cells were suspended with 100 mM acid-disodium hydrogen phosphate buffer at pH 6.2 for controlling the OD_600_ to 10 and disrupted by JY92-IIN ultrasonication (Ningbo xinzhi biotechnology, Ltd., Ningbo, China), and the lysate was centrifuged at 8000 × *g* for 10 min. The supernatant was used as a crude enzyme and was preserved at −20°C prior to use.

### Optimization Experiments of the ChBD-CadA Immobilization on Chitin

The adsorption experiments were performed in a 1 mL reaction volume containing 10 g/L chitin and 1.50 mg/mL of the crude enzyme with activity of 68.67 U/mL in a 2 mL centrifuge tube at 200 rpm stirring under various conditions.

The effects of varying temperatures (20°C, 25°C, 30°C, 35°C, and 40°C), times (2 min, 5 min, 10 min, 30 min, 60 min, and 120 min), pH (5.0, 5.6, 6.2, 6.8, 7.4, and 8.0), and protein concentrations (0.40 mg/mL, 0.80 mg/mL, 1.20 mg/mL, 1.60 mg/mL, and 2.00 mg/mL) on enzyme immobilization were investigated. The supernatant was collected after centrifugation at 6000 × *g* for 5 min at 4°C. The concentrations of protein and CadA activities before and after immobilization were assayed. Each assay was carried out in triplicate and the averages with standard deviations are presented.

### Characterization of Chitin Before and After ChBD-CadA Immobilization

Scanning electron microscopy (Hitachi S-3400, Tokyo, Japan) was conducted to investigate the immobilized ChBD-CadA on chitin. The chitin before and after ChBD-CadA immobilization was dried by an auto critical-point dryer and spread on copper grids coated with a carbon support film, followed by coating with gold prior to observation at 10 kV.

Laser scanning confocal microscopy (Zeiss LSM880, Ostalbkreis, Germany) was performed to explore the chitin before and after GFP-ChBD-CadA immobilization.

Fourier transform infrared spectra (FTIR) of chitin before and after immobilization with ChBD-CadA were performed by a Nicolet NEXUS 670 FT-IR instrument.

The elemental analysis (EA) of the chitin, ChBD-CadA, and I-ChBD-CadA were determined using a Vario EL Cube instrument (Elementar Analysensysteme GmbH, Hanau, Germany).

Measurements of Thermal gravimetric analysis (TGA) were conducted using a NETZSCH TG 209 F1 Libra thermo gravimetric analyzer. The heating rate was 10°C/min from 30°C to 800°C under nitrogen atmospheres (100 mL/min).

### Comparison of Properties of Free CadA, ChBD-CadA and I-ChBD-CadA

The optimum temperature for activity of CadA, ChBD-CadA, and I-ChBD-CadA were examined in the range of 25–65°C. For thermal stability, enzymes without substrate were incubated in 100 mM acid-disodium hydrogen phosphate buffer (pH 6.2) at 30–55°C for 2 h and the residual activities were determined.

The effect of pH on the enzymes was tested using 100 mM citric acid-disodium hydrogen phosphate buffer at pH 5.0–8.0 and 45°C. To determine pH stability, the enzymes without substrate were incubated at various pH values and 45°C for 3 h, and the residual activities were determined.

To estimate the kinetic parameters of CadA, ChBD-CadA, and I-ChBD-CadA, the initial velocities were determined by incubating 10 μg/mL purified enzyme with L-lysine concentrations ranging from 1 to 8 mM at 45°C in 1 mL reaction volume containing 100 mM citric acid hydrogen phosphate disodium buffer (pH 6.2) and 0.1 mM PLP for 20 min, and then terminated by heating at 100°C for 5 min. The determination of enzyme activity was according to the release of cadaverine.

The *K*_m_ and *V*_max_ values were obtained by Lineweaver–Burk Plots (Price, 1985), when the reaction of CadA was linearly with concentration of L-lysine (1–8 mM).

### The Batch Production of Cadaverine Using I-ChBD-CadA

To investigate the optimal substrate concentration, conversion was performed in 20 mL reaction mixture containing a final concentration of 100 mM citric acid hydrogen phosphate disodium buffer (pH 6.2), 0.1 mM PLP, and various concentrations of L-lysine (100.0, 150.0, 200.0, and 250.0 g/L) in a 200 rpm shaking incubator at 45°C for 60 min.

Batch production of cadaverine was performed with 200.0 g/L L-lysine under the same conditions. The concentration of L-lysine and cadaverine were measured at different time intervals.

The molar (M) yield of cadaverine was calculated according to the following equation:

Cadaverineyield(%) =cadaverine⁢(M)⁢produced/L-lysine⁢(M)⁢addition

### Repeated Use of the I-ChBD-CadA

The initial concentration of L-lysine (200.0 g/L) was used to test the reusability of I-ChBD-CadA. When the production rate of cadaverine slowed in each reaction, the reaction solution was centrifugation at 6000 × *g* for 10 min, and the precipitate was washed two times with ddH_2_O. The sediment was re-suspended in buffer (100 mM citric acid-disodium hydrogen phosphate buffer, pH 6.2) and then fresh substrate was fed into the bioconversion system for the next reaction.

### Analytical Method

All recombinant protein samples were analyzed by reductive sodium dodecyl sulfate polyacrylamide gel electrophoresis (SDS-PAGE) with 20 mM β-mercaptoethanol incubation. A premixed protein marker (Takara Biotechnology Co., Ltd., Nanjing, China) containing 180-, 140-, 100-, 75-, 60-, and 45-kDa protein bands was used as the molecular mass standard.

The molecular mass of recombinant proteins was calculated using the ExPASy Protparam tool.^2^ The deduced amino acid sequence of the fusion protein was used to predict the 3D structures using the RaptorX tool.^3^

The L-lysine concentration was determined using an SBA-40E immobilized enzyme biosensor (Institute of Biology, Shandong, China).

High-performance liquid chromatographic (HPLC) analysis of cadaverine was performed on an Agilent 1290 Infinity system (Agilent Technologies, Santa Clara, CA, United States) equipped with fluorescence detector (FLD G1321B; Agilent Technologies, Santa Clara, CA, United States). Specific steps were conducted according to our previous study (Ma et al., 2015b).

Protein concentrations were determined by absorption at 280 nm using the Bradford method with bovine serum albumin as the standard (Bradford, 1976).

The CadA activity assay used L-lysine hydrochloride as the substrate. A mixture (1 mL) containing 500 μL enzyme, 100 mM citric acid hydrogen phosphate disodium buffer (pH 6.2), 0.1 mM PLP, and 450 mM (100.0 g/L) L-lysine was incubated at 45°C and 200 rpm was incubated for 20 min, and then terminated by heating at 100°C for 5 min. One unit of CadA activity (U) was defined as the amount of enzyme required to produce 1 mmol cadaverine per minute at 45°C and pH 6.2.

## Results and Discussion

### Construction and Expression of Fusion Gene

Four expression plasmids pETDuet-*CadA*, pET28a(+)-*ChBD-CadA*, pET28a(+)-*CadA-ChBD*, and pET28a (+)-*gfp-ChBD-CadA*, containing the sequences of the recombinant CadA, ChBD-CadA, CadA-ChBD, and GFP-ChBD-CadA, respectively, were constructed and successfully transformed into *E. coli* BL21(DE3) cells.

The expression of four genes was analyzed by SDS-PAGE. As shown in Supplementary Figure S1, the crude enzyme of *E. coli* BL21(DE3) harboring pET28a(+)-*CadA-ChBD* shows no target band (lane 3), which suggests *CadA-ChBD* cannot be expressed in *E. coli* BL21(DE3). *E. coli* BL21(DE3) harboring the pETDuet-*CadA*, pET28a(+)-*ChBD-CadA*, and pET28a(+)-*gfp-ChBD-CadA* indicates clear bands at approximately 80.0 kDa (lane 2) for CadA, 95.0 kDa (lane 4) for ChBD-CadA, and 119.0 kDa (lane 5) for GFP-ChBD-CadA. These sizes are in agreement with those calculated from the amino acid sequence of CadA (80.4 kDa), ChBD-CadA (96.2 kDa), and GFP-ChBD-CadA (∼119.4 kDa), respectively, and correspond to those of ChBD (∼15.3 kDa) and CadA (∼80.5 kDa)(Park et al., 2017) and GFP (∼24.1 kDa) (Zhang et al., 2017).

The optimal temperature of CadA expression in *E. coli* BL21(DE3) was 37°C, which was consistent with other reports and our previous studies (Ma et al., 2015a; Seo et al., 2016). However, ChBD-CadA formed inclusion bodies at 37°C, and its optimal temperature of expression was 30°C in this study (data not shown). Meanwhile, the expression level of ChBD-CadA decreased by around 1/3 (68.67 U/mL crude enzyme) compared to that of CadA (99.57 U/mL crude enzyme) according to analysis of SDS-PAGE and CadA activity (Supplementary Figure S1 and Supplementary Table S2). These results suggested that the fusion of ChBD affected the expression of CadA gene. Other studies also reported that the gene expression will decline after fusion of ChBD or other genes (Pham et al., 2017), which are similar with this study.

### Simultaneous Purification and Immobilization of ChBD-CadA by Chitin

**FIGURE 1:**
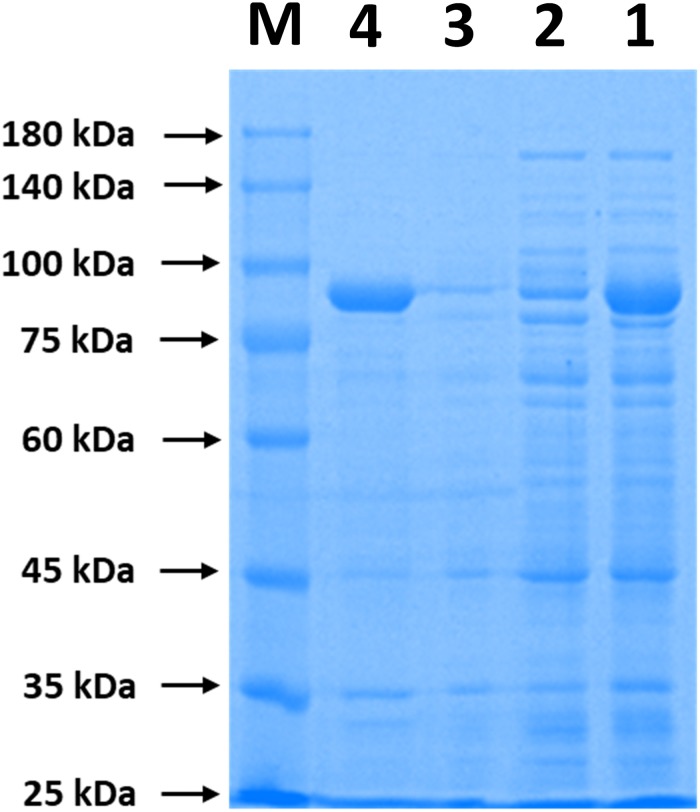
SDS-PAGE analysis of ChBD-CadA immobilization. Lane M, protein Marker; lane 1, the crude enzyme of ChBD-CadA; lane 2, the crude enzyme of ChBD-CadA after chitin adsorption; lane 3, rinsing fractions of complex of chitin of ChBD-CadA immobilized. lane 4, the eluent of the ChBD-CadA immobilized on chitin by SDS (1.0 M). The adsorption condition was conducted with chitin at 10 g/L and 25°C for 30 min at pH 6.8 and a protein concentration of 1.60 mg/mL.

The immobilization of ChBD-CadA on chitin was analyzed by SDS-PAGE. As shown in Figure 1, the band of ChBD-CadA can be seen in lanes of crude enzyme (lane 1) and was significantly reduced after chitin adsorption (lane 2). Meanwhile, the single band of ChBD-CadA was obtained after SDS elution (lane 4). These results suggest that the majority of ChBD-CadA can be efficiently bound to chitin due to the high specific affinity of ChBD for chitin. Other studies also reported that ChBD possesses significant binding affinity for various enzyme immobilizations (Pham et al., 2017; Xu et al., 2017).

**FIGURE 2:**
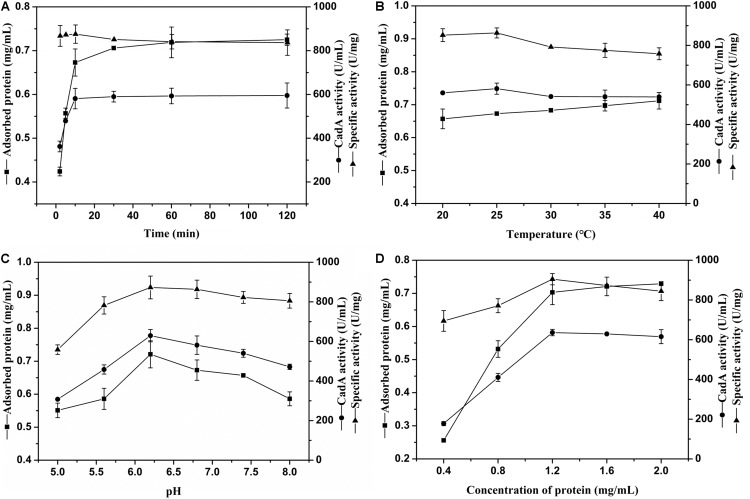
**(A)** The effect of time on CadA adsorption (temperature, 25°C; pH, 6.8; protein concentration, 1.50 mg/mL). **(B)** The effect of temperature on CadA adsorption (time, 10 min; pH, 6.8; protein concentration, 1.50 mg/mL). **(C)** The effect of pH on CadA adsorption (time, 10 min; temperature, 25°C; protein concentration, 1.50 mg/mL); **(D)** The effect of protein concentration on CadA adsorption (time, 10 min; temperature, 25°C; pH, 6.2). Data listed were performed in triplicate and reported as the average with standard deviations for assays.

The external environment will have various effects on enzyme immobilization (Huang et al., 2018). The relevance between chitin and immobilization time is shown in Figure 2A. The ChBD-CadA was almost completely immobilized on chitin within 10 min with a maximum specific activity of 86.33 U/mg, which was consistent with our previous result of chitinase (*Cm*Chi1) adsorption by chitin. Skujins et al. (1973) reported the adsorption reaction between chitin and ChBD in chitinase was very fast and was nearly accomplished within 2 min (Skujins et al., 1973). With extended adsorption time, the concentration of protein adsorbed didn’t obviously increase, nor did the specific activity or CadA activity. These observations may be the result of an adsorption equilibrium of chitin being established at 10 g/L.

The adsorption of ChBD-CadA on chitin at various temperatures is compared in Figure 2B. The results show the protein adsorption increased with increasing temperature. The CadA activity adsorbed from 20–40°C possessed no obvious differences to the maximum reached at 25°C (58.13 U/mL) with specific activity of 86.33 U/mg. A previous report indicated that temperature of 10–25°C was favorable for the ChBD of chitinase adsorption on chitin (Pang et al., 2007). Thus, 25°C was chosen for subsequent experiments due to the optimal adsorption and specific activity.

The effect of various pH values on ChBD-CadA immobilization is illustrated in Figure 2C. Both the concentration of protein and immobilized CadA activity increased from pH 5.0 to 6.2 and decreased after pH 6.2. The maximum concentration of protein (0.72 mg/mL) and immobilized CadA activity (62.93 U/mL) was obtained at pH 6.2, with the highest specific activity of 87.28 U/mg. This result suggested that the affinity adsorption between chitin and ChBD-CadA was most favorable under weak acidic conditions. The phenomenon could be explained that the chitinase (*Cm*Chi1) showed a better activity at weak acidic conditions (Zhang et al., 2018). Thus, the ChBD from *Cm*Chi1 also possessed a similar property.

As shown in Figure 2D, the protein concentration absorbed was enhanced as the protein concentration increased. The absorbed CadA activity also increased from 17.78 U/mL to 63.62 U/mL relative to a shift in protein concentration from 0.40 mg/mL to 1.20 mg/mL. Following this increase, the protein concentration and CadA absorbed remained constant, showing that the optimized concentration of protein using chitin of 10 g/L as carrier was 1.20 mg/mL.

Based on the above results, the optimal immobilized conditions were as follows: temperature, 25°C; time, 10 min; pH, 6.2; protein concentration, 1.20 mg/mL, which can bind with CadA activity of 68.67 U/mL and 93% immobilized ratio. Pham et al. (2017) also reported that the immobilized ratio of fusion protein can reach 99% and 91.0% using the ChBD of chitinase A1, respectively.

### Characterization of Immobilized Enzyme on Chitin

**FIGURE 3:**
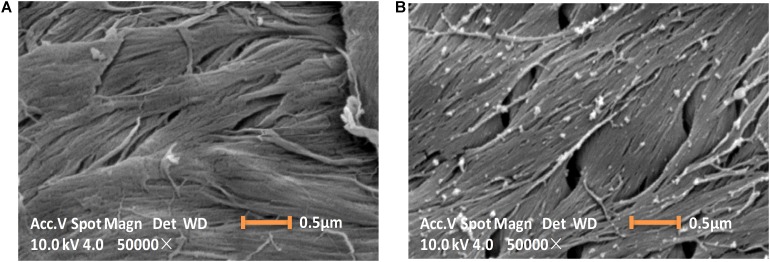
Scanning electron images (10 KV, 50000×) showing the morphology image of chitin before **(A)** and after **(B)** ChBD-CadA immobilization.

The ChBD-CadA immobilization on chitin was confirmed by scanning electron microscopy, as shown in Figure 3. The chitin without immobilized enzyme presented as a fibrous and porous structure. Some aggregates of protein (ChBD-CadA) were observed on the surface of chitin after immobilization. Our previous study also showed that the protein molecules were clearly found on the surface of chitin in the affinity adsorption of chitinase containing ChBD on chitin (Zhang et al., 2016).

**FIGURE 4:**
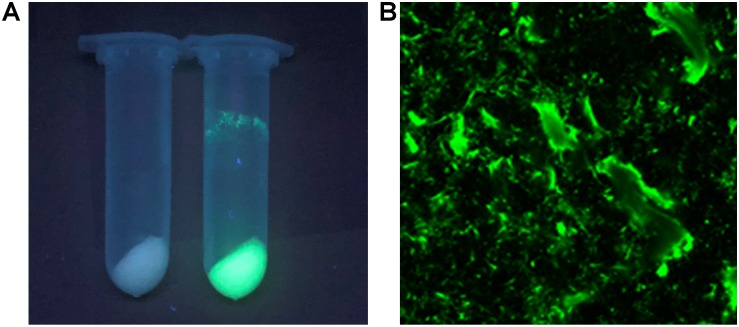
**(A)** Photograph of chitin under the UV lamp (365 nm) before and after enzyme adsorption. Samples (2 mL) were centrifuged at 12,000 r/min. **(B)** Laser scanning confocal microscope image (excitation: 488 nm, emission: 507 nm, at 25 × magnification) of chitin powder after ChBD-CadA adsorption.

The I-ChBD-CadA was also investigated with laser scanning confocal microscopy via fusion with GFP. As shown in Figure 4A, chitin appears white without green fluorescence and green fluorescence was apparent under a UV lamp before and after GFP-ChBD-CadA immobilization. In addition, laser scanning confocal microscopy was used to assess the GFP-ChBD-CadA immobilization. No green fluorescence was present in chitin before the enzyme immobilization (Supplementary Figure S2), and the surface of chitin showed significant green fluorescence after enzyme immobilization (Figure 4B).

Fourier transform infrared spectra of chitin, ChBD-CadA, and I-ChBD-CadA were studied (Supplementary Figure S3). The characteristic absorption bands of chitin were at 1554 cm^–1^ (amide II bond), 1619 cm^–1^, and 1655 cm^–1^ (amide I, single H-bond, and double H-bond, respectively), 3255 cm^–1^ (N-H-stretching) and 3424 cm^–1^ (O-H-stretching band) (J. Brugnerotto et al., 2001). ChBD-CadA possessed characteristic peaks at 1554 cm^–1^ and 1660 cm^–1^, which correspond to the stretching vibration of the protein-specific amide II bond (-NH-) and amide II bond (C = O), respectively (Liang et al., 2015). After immobilization, the peaks (1619 cm^–1^ and 1655 cm^–1^) of chitin were covered by peak (1660 cm^–1^) of ChBD-CadA, which indicated that ChBD-CadA had been immobilized on chitin.

The TGA curves of CadA, ChBD-CadA, and I-ChBD-CadA are shown in Supplementary Figure S4. The TGA curve of I-ChBD-CadA was different from that of chitin but shared similar tendency with ChBD-CadA. For chitin, a weight loss of ∼5% was observed in the range 30–110°C due to the evaporation of water (Kaya et al., 2016). While in the range 280–400°C, most of the weight loss has been accomplished, leading to a carbonaceous material residue (Qiao et al., 2015). In the range 30–200°C, the weight of ChBD-CadA and I-ChBD-CadA slowly declined about 14wt% and 12wt% because of the removal of water. In the range 200–270°C, ChBD-CadA had an obviously weight loss (∼10wt%), as well as I-ChBD-CadA, which indicated the decomposition of the enzyme molecule.

The elemental analysis of chitin and I-ChBD-CadA shows that the C, H, N of I-ChBD-CadA are 41.52%, 6.42%, and 6.55%, respectively, which are between that of chitin and ChBD-CadA (Supplementary Table S3). These results also confirmed the effective immobilization of the ChBD-CadA on chitin.

### The Effect of ChBD on 3D Structure of CadA

The 3D structure prediction of CadA was investigated in this study. The prediction showed that the 3D structure of CadA was a decamer (Supplementary Figure S5A) in its active structure, which agreed with other reports (Kanjee et al., 2011). The predictions of ChBD and CadA monomer structures are shown in Supplementary Figure S5B and Supplementary Figure S5C, respectively. The 3D structure of ChBD-CadA showed the structures of ChBD and CadA were not significantly different than before the fusion. The result can be explained by the sequence of 4 residues (VVGG) in the C-terminus of ChBD, and the restriction sites (KL) between ChBD and CadA might be a flexible linkage between ChBD to CadA. Meanwhile, the prediction indicated that the two ChBD domains were both arranged well separated from the CadA monomer (located at the right and inner), whether viewed from the front (Supplementary Figure S5D) or from a rotation of 90° (Supplementary Figure S5E). This indicates that the ChBD domain is free to maintain the original adsorption function while in associated with CadA, and at the same time does not affect the formation of CadA decamer (active form), which are in agreement with the results of this study.

The binding mechanism of ChBD-CadA on chitin was also studied. The amino acids residues (His31, Thr32, Trp39, Trp126, and Trp127) in the functional domains of ChBD from *Cm*Chi1 are highly conserved, compared with that of reported ChBD (Hashimoto et al., 2000). Among, these residues (His31, Thr32, Trp39, and Trp127) are located on one face of the conformation and are much exposed to the chitin surface (Supplementary Figure S6), which are proposed to bind with the GlcNAc residues of chitin chains through hydrophobic and π/π interactions (Bernard et al., 2004; Kikkawa et al., 2014).

### The Comparison of CadA, ChBD-CadA, and I-ChBD-CadA

**FIGURE 5:**
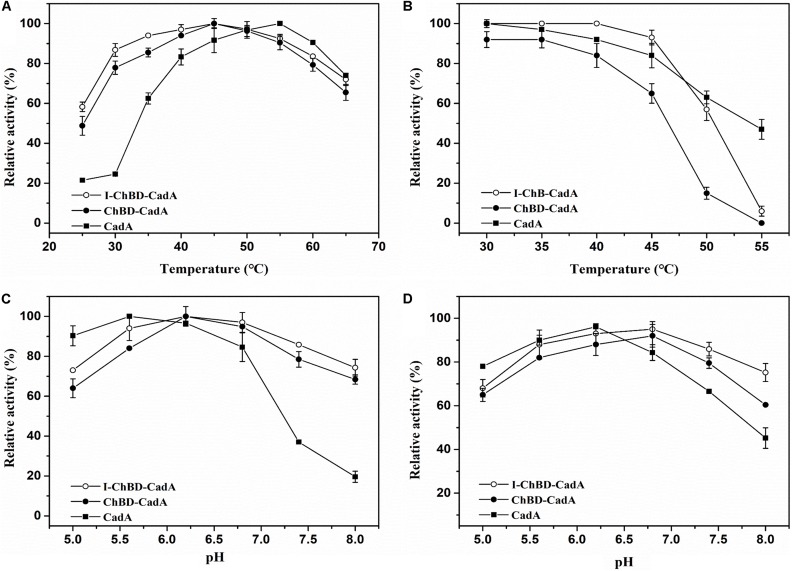
**(A)** The optimum temperature for CadA (■), ChBD-CadA (•), and I-ChBD-CadA (○). The optimal temperature was performed at various temperatures under their optimum pH (100 mM acid-disodium hydrogen phosphate buffer, pH 5.6 for CadA, pH 6.2 for free and immobilized ChBD-CadA). Relative activity was expressed as a percentage of maximum activity (99.57 U/mL for CadA and 68.67 U/mL for both free and immobilized ChBD-CadA) under the reaction conditions. **(B)** The temperature stability for CadA (■), ChBD-CadA (•), and I-ChBD-CadA (○). To determine the thermostability, the residual activity was measured in their optimum pH (same as in Figure 7A legend) after the enzyme was treated for 3 h at different temperatures. The original activity without pre-incubation was taken to be 100% (87.54 U/mL for wild-type free CadA and 61.33 U/mL for both free and immobilized ChBD-CadA). **(C)** The optimum pH for CadA (■), ChBD-CadA (•), and I-ChBD-CadA (○). The optimum pH was measured at 45°C in 100 mM acid-disodium hydrogen phosphate buffer with various pH values. Relative activity was expressed as a percentage of maximum activity (104.22 U/mL for wild-type free CadA, 84.79 U/mL for free and immobilized ChBD-CadA) under the reaction conditions. **(D)** The pH stability for CadA (■), ChBD-CadA (•), and I-ChBD-CadA (○). The optimum pH was incubated at 45°C for 3 h in 100 mM acid-disodium hydrogen phosphate buffer with various pH values. Residual activity was expressed as a percentage of the initial activity measured without pre-incubation.

The effect of temperature on the activity of CadA, ChBD-CadA, and I-ChBD-CadA were next examined. As shown in Figure 5A, the optimal temperature of CadA was 55°C, which agreed well with a previous report (Kou et al., 2018). The free ChBD-CadA showed an optimal temperature at 45°C, which demonstrated that the optimal temperature of CadA changed after fusion with ChBD. In addition, immobilization of ChBD-CadA did not affect its optimal temperature, but increased the temperature arrange.

For prolonged enzyme reactions, the thermal stability was tested. As shown in Figure 5B, the temperature stability of CadA decreased after fusion with ChBD. However, the immobilization of ChBD-CadA enhanced its temperature stability, especially at temperatures above 50°C.

The effect of pH on the CadA, ChBD-CadA, and I-ChBD-CadA were also tested, and the results can be seen in Figure 5C. The optimal pH of CadA was 5.6, and the activity dropped quickly when pH > 7.5, which was consistent with the previous study (Kou et al., 2018). The phenomenon may be the result of a loss of quaternary structure in which the decamer of CadA (high activity) transforms to the dimer structure (low activity) at pH > 7.5. ChBD-CadA and immobilized ChBD-CadA both showed maximum activity at pH 6.2, but maintain more than 73% of their highest activity at pH 8.0, which showed that the alkali resistance of CadA was improved after fusion with ChBD. Meanwhile, the pH range of ChBD-CadA showed no obvious change after immobilization.

Compared to CadA, the stability of ChBD-CadA was improved from pH 6.8 to 8.0 (Figure 5D). In addition, immobilization of ChBD-CadA can further enhance its pH stability; more than 75% of the highest activity can be obtained at pH 8.0.

The kinetic parameters of CadA, ChBD-CadA and I-ChBD-CadA for L-lysine were provided (Table 1). *V*_max_, *K*_m_, and *K*_cat_ of ChBD-CadA were 23.04 nmol/min/μg, 0.66 mM, and 0.13 min^–1^, respectively, which were similar with that of CadA. The result suggested that the fusion of ChBD possessed little effect on the activity of CadA. However, the *V*_max_ (14.57 nmol/min/μg) and *K*_cat_ (0.07 min^–1^) of I-ChBD-CadA were lower than that of ChBD-CadA, which indicated that the activity of ChBD-CadA decreased after immobilization. Pham et al. (2017) also found the activity of β-galactosidases declined after immobilization on chitin, which was similar with our study.

**TABLE 1:** Kinetic parameters of CadA, ChBD-CadA, and I-ChBD-CadA.

**Kinetic parameter**	**CadA**	**ChBD-CadA**	**I-ChBD-CadA**
*V*_max_ (nmol product/min/μg)	24.57 ± 1.16	23.04 ± 1.32	14.57 ± 0.08
*K*_m_ (mM)	0.64 ± 0.03	0.66 ± 0.08	0.81 ± 0.17
*K*_cat_ (min^–1)^	0.13 ± 0.01	0.12 ± 0.01	0.07 ± 0.01

### The Batch Production of Cadaverine Using Immobilized ChBD-CadA

**FIGURE 6:**
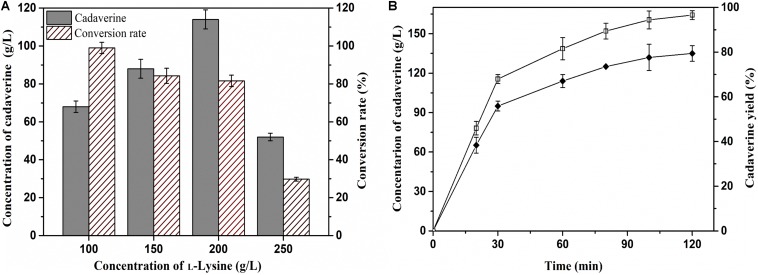
**(A)** The effect of L-lysine initial concentration on cadaverine production. **(B)** The batch production of cadaverine using I-ChBD-CadA.

Achieving a high concentration of product is very important in the industrial production of chemicals (Guo et al., 2018). Thus, the effects of L-lysine concentration on batch production of cadaverine using immobilized ChBD-CadA were first evaluated. Cadaverine increased from 68.3 g/L to 114.5 g/L as the concentration of L-lysine increased from 100.0 to 200.0 g/L along with a weak decline of yield from 98 to 84%, as shown in Figure 6A. With the further increase of L-lysine (250.0 g/L), the concentration and yield both decreased significantly to 50.2 g/L and 25%, respectively, which suggests that the immobilized ChBD-CadA exhibited apparent substrate inhibition at high concentrations of L-lysine. Thus, the initial concentration of L-lysine (200.0 g/L) was used to study the batch production of cadaverine using immobilized ChBD-CadA. As shown in Figure 6B, the concentration of cadaverine increased rapidly in the first 30 min, and was followed by a gradual decrease in production intensity. This result may be explained by the CadA activity being inhibited at high concentrations of cadaverine. Finally, a cadaverine concentration of 135.6 g/L with a molar yield of 97% was achieved in 120 min. Especially, the conversion can be conducted without pH control, compared with other reports (Park et al., 2015; Kloss et al., 2018). In their studies, the production of cadaverine by whole-cell biotransformation need extra acid to maintain the pH around 6.0. The reason can be explained that immobilized ChBD-CadA maintained good activity under the final pH (8.0) as described above.

### The Repeated Use of Immobilized ChBD-CadA

**FIGURE 7:**
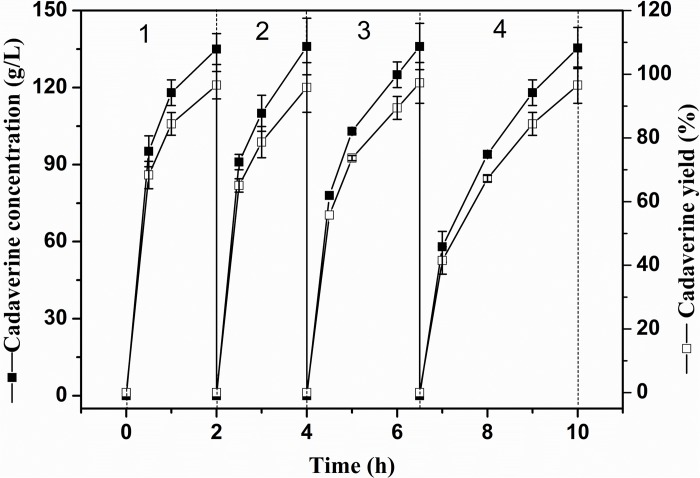
The repeated use of I-ChBD-CadA.

The reusability of immobilized enzymes plays an important role in industrial bioconversions (Sheldon and van Pelt, 2013). Thus, the reusability of the I-ChBD-CadA was determined using L-lysine at 200.0 g/L. As shown in Figure 7, the concentration of cadaverine increased over time and the substrate was almost completely transformed to cadaverine with approximately 95–97% yield every batch. From cycle 1 to cycle 4, the time to completely convert was gradually increased from 2 to 3.5 h, along with the decline of residual enzyme activity from 65.40 U/mL to 39.14 U/mL. However, the residual protein maintained similar amount (Table 2). These results indicated that the loss of enzyme activity after reuse led to the extension of conversion time from cycle 1 to cycle 4, not the release of ChBD-CadA from chitin. Consequently, an average concentration of cadaverine (135.1 g/L) was obtained within 10 h and an activity of 57% was retained through four cycles, and a total of 540.4 g of cadaverine in 4 L conversion reactions was achieved. Seo et al. (2016) reported a 75–80% conversion yield over five reaction cycles by fusion with phasin immobilization of CadA on intracellular PHA (Seo et al., 2016). Park et al. (2017) reported that a 53% residual activity was obtained after the 10th recycle using cross-linked enzyme aggregates of CadA (Park et al., 2017). These results demonstrated that the immobilization of CadA on chitin permits enzyme reuse. However, the concentrations of cadaverine used in their studies were both far lower than that of this study.

**TABLE 2:** Results of the I-ChBD-CadA reuse.

**Cycle times**	**Time (h)**	**Cadaverine yield (%)**	**Residual enzyme activity (U/mL)**	**Residual protein amount (mg/mL)**	**Specific activity (U/mg)**
1	2	97.23 ± 2.12	65.40 ± 1.48	0.71 ± 0.05	92.12 ± 3.2
2	2	95.25 ± 1.89	61.04 ± 3.10	0.70 ± 0.03	87.28 ± 4.5
3	2.5	96.30 ± 1.91	51.79 ± 2.58	0.67 ± 0.06	77.30 ± 3.7
4	3.5	97.11 ± 2.47	37.28 ± 1.09	0.64 ± 0.04	58.25 ± 1.1

The microscopy surface of chitin after repeated use was also investigated. As shown in Supplementary Figure S7, the surface of chitin after reuse maintained an original structure, which suggested that the chitin possesses a good stability.

These results showed that the immobilized ChBD-CadA on chitin for cadaverine enzymatic production is feasible and possesses potential industrial application for cadaverine production.

## Conclusion

In this study, chitin was used as the carrier for efficient CadA immobilization via fusion with ChBD for the production of cadaverine from L-lysine. The ChBD-CadA fusion protein showed better pH stability compared with wild type of CadA. Further, it was capable of being directly immobilized from crude enzyme with CadA activity of 93% under optimal conditions, and the I-ChBD-CadA was used to convert L-lysine at 200.0 g/L, achieving 135.6 g/L of cadaverine with a 97% molar yield. In addition, the I-ChBD-CadA can be reused in high substrate concentration without the addition of any acids. This is first report of lysine decarboxylase immobilization via ChBD fusion aimed at the industrial production of cadaverine. The process provides the others to design similar immobilization with various applications.

## Data Availability Statement

The datasets generated for this study are available on request to the corresponding author.

## Author Contributions

PO, KC, and SX conceived and designed the research. NZ and SY performed the experiments. NZ, AZ, and GW analyzed the data. NZ and KC wrote the manuscript. All authors commented on the manuscript and approved the contents.

## Conflict of Interest

The authors declare that the research was conducted in the absence of any commercial or financial relationships that could be construed as a potential conflict of interest.

## Footnotes

1 http://www.snapgene.com/

2 http://web.expasy.org/protparam/

3 http://raptorx.uchicago.edu/
