# Supplementary material for: Cadaverine Production From L-Lysine With Chitin-Binding Protein-Mediated Lysine Decarboxylase Immobilization
Source: Front Bioeng Biotechnol. 2020 Mar 3;8:103. doi: 10.3389/fbioe.2020.00103 (PMC7062646; doi:10.3389/fbioe.2020.00103)
Supplement: Supplementary file 1 [file Table_1.DOCX]

Supplementary Material

Table S1. Oligonucleotide primers used for PCR

| Target gene | Primer | Sequence (5’-3’) |
| --- | --- | --- |
| *CadA* | F1 (HindIII) | CCCAAGCTTCAATGGATGAACGTTATTGCAATAT |
|  | R1 (NotI) | ATAAGAATGCGGCCGCAATACCTTAACGGTATAGCGG |
|  | F2 (NcoI) | CATGCCATGGATGGATGAACGTTATTGCAATAT |
|  | R2 (BamHI) | CGGGATCCTTCTTTCAATACCTTAACGG |
| *ChBD* | F3 (BamHI) | CGCGGATCCATGTACCCGGTATGGCAG |
|  | R3 (HindIII) | CCCAAGCTTCGGCGTCGGAGTAGCAACAACC |
| *gfp* | F4 (NdeI) | GGAATTCCATATGATGGTGAGCAAGGGC |
|  | R4 (BamHI) | CGGGATCCCTTGTACAGCTCGTCCAT |

Table S2. The effect of ChBD fusion on expression and activity of CadA

| Protein | CadA activity  (U/mL crude enzyme^a^) | Protein concentration  (mg/mL) | Specific activity  (U/mg total protein) |
| --- | --- | --- | --- |
| CadA | 99.57±5.32 | 1.80 | 52.29±3.21 |
| ChBD-CadA | 68.67±1.85 | 1.50 | 45.78±2.69 |

^a^The crude enzyme was obtained from the same cell density as described in the experimental section. Each experiment was assayed in triplicate and the average values with standard deviations are presented.

Table S3. Elemental analysis of chitin before and after immobilization

| Sample name | Elemental content (%) | | |
| --- | --- | --- | --- |
|  | C | H | N |
| Chitin | 42.92 | 6.58 | 6.42 |
| ChBD-CadA | 21.80 | 4.59 | 7.21 |
| I-ChBD-CadA | 41.52 | 6.42 | 6.55 |





Figure S1. SDS-PAGE analysis of expressions of CadA, CadA-ChBD, ChBD-CadA, and GFP-ChBD-CadA gene. The amount of protein applied to the gel is 20 μg. Lane M, protein Marker; lane 1; cell-free extracts of *E. coli* BL21(DE3); lane 2, cell-free extracts of *E. coli* BL21(DE3) harboring CadA gene; lane 3, cell-free extracts of *E. coli* BL21(DE3) harboring CadA-ChBD gene; lane 4, cell-free extracts of *E. coli* BL21(DE3) harboring ChBD-CadA; lane 5, cell-free extracts of *E. coli* BL21(DE3) harboring GFP-ChBD-CadA gene.





Figure S2. Laser scanning confocal microscope image (excitation: 488 nm, emission: 507 nm, at 25x magnification) of chitin powder without ChBD-CadA adsorbed.





Figure S3. FTIR spectra of chitin (black line), ChBD-CadA (red line) and I-ChBD-CadA (blue line).





Figure S4. TGA curves of chitin (black line), ChBD-CadA (red line) and I-ChBD-CadA (blue line)





Figure S5. a) Predicted 3D structures of CadA decamer. b) CadA monomer. c) ChBD from *Cm*Chi1. d) ChBD-CadA. (e) ChBD-CadA after rotation by 90°.





Figure S6. Schematic illustration of the binding mechanism between chitin and ChBD used.





Figure S7. Scanning electron microscopy of chitin after repeated use
